# Estradiol Attenuates Ischemia-Induced Death of Hippocampal Neurons and Enhances Synaptic Transmission in Aged, Long-Term Hormone-Deprived Female Rats

**DOI:** 10.1371/journal.pone.0038018

**Published:** 2012-06-04

**Authors:** Tomoko Inagaki, Naoki Kaneko, R. Suzanne Zukin, Pablo E. Castillo, Anne M. Etgen

**Affiliations:** Dominick P. Purpura Department of Neuroscience, Albert Einstein College of Medicine, Bronx, New York, United States of America; University of Bristol, United Kingdom

## Abstract

**Background:**

Transient global forebrain ischemia causes selective, delayed death of hippocampal CA1 pyramidal neurons, and the ovarian hormone 17β-estradiol (E2) reduces neuronal loss in young and middle-aged females. The neuroprotective efficacy of E2 after a prolonged period of hormone deprivation is controversial, and few studies examine this issue in aged animals given E2 treatment after induction of ischemia.

**Methodology/Principal Findings:**

The present study investigated the neuroprotective effects of E2 administered immediately after global ischemia in aged female rats (15–18 months) after 6 months of hormone deprivation. We also used electrophysiological methods to assess whether CA1 synapses in the aging hippocampus remain responsive to E2 after prolonged hormone withdrawal. Animals were ovariohysterectomized and underwent 10 min global ischemia 6 months later. A single dose of E2 (2.25 µg) infused intraventricularly after reperfusion significantly increased cell survival, with 45% of CA1 neurons surviving vs 15% in controls. Ischemia also induced moderate loss of CA3/CA4 pyramidal cells. Bath application of 1 nM E2 onto brain slices derived from non-ischemic aged females after 6 months of hormone withdrawal significantly enhanced excitatory transmission at CA1 synapses evoked by Schaffer collateral stimulation, and normal long-term potentiation (LTP) was induced. The magnitude of LTP and of E2 enhancement of field excitatory postsynaptic potentials was indistinguishable from that recorded in slices from young rats.

**Conclusions/Significance:**

The data demonstrate that 1) acute post-ischemic infusion of E2 into the brain ventricles is neuroprotective in aged rats after 6 months of hormone deprivation; and 2) E2 enhances synaptic transmission in CA1 pyramidal neurons of aged long-term hormone deprived females. These findings provide evidence that the aging hippocampus remains responsive to E2 administered either in vivo or in vitro even after prolonged periods of hormone withdrawal.

## Introduction

Transient global or forebrain ischemia during cardiac arrest affects 150,000 Americans each year, most of them elderly [1], and in many cases results in selective, delayed death of hippocampal CA1 neurons [2] and severe cognitive deficits [3]. Although basic research provides solid evidence that the ovarian hormone 17β-estradiol (E2) exerts profound neuroprotective effects in young and middle-aged female and in young male rodents [4], whether E2 retains its neuroprotective actions against ischemia-induced brain damage in aging hippocampus after a prolonged period of hormone withdrawal is controversial.

It has been proposed that there is limited time window for E2 to retain its beneficial effects after loss of ovarian function due to surgical or natural menopause. The existence of this time window for beneficial actions of E2 was originally reported in an animal study [5], and was then proposed to explain why large clinical trials, such as the Women’s Health Initiative and the Women Estrogen Stroke Trial, reported that hormone therapy (HT) did not reduce the incidence of cardiovascular and neurodegenerative diseases or cognitive impairment in elderly women [6], [7], [8]. Indeed, these studies indicated that HT may increase the risk of stroke and dementia in postmenopausal women who initiated treatment long after menopause [9]. Subsequent studies of animals undergoing focal and global ischemia provided further evidence for a limited time window for E2 neuroprotection. One week pretreatments that maintain low physiological E2 levels (10–25 pg/ml) protect young mice from focal ischemia [10] and young and middle-aged rats [11] from global ischemia-induced brain damage when initiated immediately after ovariectomy, but not when initiated 10 weeks later. E2 pellets that maintain high physiological hormone levels (60–80 pg/ml) exacerbate focal ischemia-induced infarcts in reproductively senescent, middle-aged females [12], [13]. Similarly, one week treatment with proestrous levels of E2 (64 pg/ml) initiated at the time of ovariectomy is protective after global ischemia in young and middle-aged rats, but not in 24 month-old females, which are likely to have ceased normal estrous cycles for many months [14].

Nonetheless, there is evidence that under some circumstances E2 retains its neuroprotective action after long-term loss of ovarian function. A single high dose of E2 (4 mg/kg) administered 30 min before global ischemia prevents neuronal loss in 18 month-old gerbils ovariectomized for two weeks to the same extent as in young and middle-aged females [15]. Similarly, two-week pretreatment with high physiological doses of E2 in middle-aged females ovariohysterectomized (OVX) for 0, 1 or 8 weeks ameliorates ischemia-induced hippocampal neuron death in all rats regardless OVX interval [16]. One week hormone replacement also reduces focal ischemia-induced brain damage in reproductively senescent, middle-aged females [17], [18]. Moreover, a high dose of E2 administered either systemically or intraventricularly immediately after global ischemia is neuroprotective in middle-aged females 2 months after OVX [19]. These findings suggest that the dose, timing and type of E2 administration may be critical factors.

The present study addressed unanswered, yet important questions about the efficacy of E2 treatment initiated after an ischemic event in aged female rats subjected to long-term loss of ovarian function. The investigation of acute post-ischemic E2 administration in old animals is of great clinical relevance as the incidence of stroke increases with age, specifically in women after menopause [20]. Moreover, treatment that can be administered after the onset of ischemia is more clinically relevant than pretreatment. Therefore, we tested the neuroprotective effects of E2 administered immediately after global ischemia in aged female rats after 6 months of hormone deprivation. In addition, we used electrophysiology to assess whether CA1 synapses in the aging hippocampus remain responsive to E2 in age-matched, non-ischemic females 6 months after OVX.

## Materials and Methods

### Animals and Ethics Statement

All experiments were conducted in accordance with the National Institutes of Health Guidelines for Care and Use of Animals in Research and were approved by the Institutional Animal Care and Use Committee at Albert Einstein College of Medicine. Middle-aged (retired breeders, 9–11 months) and young (2 months) female Sprague-Dawley rats were obtained from Charles River (Wilmington, DE) and housed in groups of 2–3. Retired breeders were chosen because the majority of middle-aged women have had at least one pregnancy. Thus, retired breeders are more clinically relevant than virgin females. All rats were bilaterally OVX under isoflurane anesthesia (4% induction, 2% maintenance in 70% N_2_O:30% O_2_). Young rats were used only for slice studies and were OVX for 7–10 days before experimentation.

### Body Weight

Because E2 reduces food intake and long–term hormone withdrawal increases body weight [21], [22], OVX animals were weighed every month after OVX. Body weight was also measured before and after ischemia/sham-operation, and again when rats were killed for histological analysis.

### Global Ischemia

Rats were subjected to transient global ischemia by four vessel occlusion or sham surgery as previously described [23]. Briefly, rats were deeply anesthetized with isoflurane, and the vertebral arteries were occluded bilaterally to prevent collateral blood flow to the forebrain during the subsequent occlusion of the common carotid arteries. One day later, transient global ischemia was induced by bilateral occlusion of the carotid arteries for 10 min. Pupil dilation and the loss of righting reflex were monitored every minute during carotid occlusion to verify ischemia. Rectal temperature was maintained at 36.5–37.5°C until recovery from anesthesia. Sham-operated rats underwent all procedures except carotid artery occlusion. Of the 48 rats that underwent global ischemia or sham surgery 6 months after OVX, 13 died during or after surgery, and 3 were excluded because they either failed to show neurological signs of ischemia or awakened during occlusion.

### Hormone Treatment

E2 was dissolved in vehicle (100% DMSO) at a dose previously shown to reduce CA1 cell death in young and middle-aged females [19]. E2 or vehicle was administered immediately after reperfusion. A 26 ga needle was lowered into the right lateral ventricle using standard stereotaxic procedures. Vehicle or 2.25 µg of E2 in 5 µl of DMSO was infused at a flow rate of 5 µl/min. For subcutaneous injection, E2 was dissolved in 100% ethanol at 10 mg/ml, then further diluted in peanut oil. A final dose of 100 µg/kg of E2 was injected at the time of reperfusion. This dose is neuroprotective in young and middle-aged, OVX females [19], [24].

### Histological Analysis

One week after global ischemia or sham surgery, rats were transcardially perfused with 0.9% saline followed by ice cold 10% phosphate buffered formalin (Fisher Scientific, Pittsburgh, PA). Brains were removed, placed in formalin at 4°C overnight, fixed in 30% sucrose in phosphate buffered saline at 4°C for 5 days and then frozen at −80°C. Coronal sections (20 µm) were cut at the level of the dorsal hippocampus (3.3–4.0 mm posterior from bregma), and 4 sections (at 140 µm intervals) per animal were mounted and stained with haematoxylin and eosin. Medial, middle, and lateral sectors (250×250 µm each) from the CA1, and one sector each from the CA3 and CA4 regions of the left and right hippocampus, were photographed at 40× magnification using an Olympus microscope and digital camera. Digital images were opened in Adobe Photoshop, and the number of viable pyramidal neurons in each region of interest was counted. Surviving neurons had rounded cell bodies and clearly visible nucleoli. Pyknotic and shrunken neurons were not counted. CA1 cell counts in each sector (medial, middle and lateral) were summated over right and left hemispheres to calculate the total number of surviving CA1 pyramidal neurons in all 4 sections for each of the 3 sectors. Similarly, surviving pyramidal neurons in left and right CA3 and CA4 were summed in each subfield. All cell counts were carried out by an investigator who was blind to the animals’ treatment.

### Electrophysiological Recordings

OVX rats were anesthetized with isoflurane and decapitated, and the brains were removed into chilled cutting solution consisting of (in mM) 215 sucrose, 2.5 KCl, 20 glucose, 26 NaHCO_3_, 1.6 NaH_2_PO_4_, 1 CaCl_2_, 4 MgCl_2_, and 4 MgSO_4_. Transverse slices (400 µm) of dorsal hippocampus were prepared with a Leica VT1200 S (Leica, Nussloch, Germany) vibrating microslicer and stored in holding solution (50% cutting and 50% recording solution), which was gradually replaced within 1 h with extracellular recording solution (aCSF) containing the following (in mM): 124 NaCl, 2.5 KCl, 10 glucose, 26 NaHCO_3_, 1 NaH_2_PO_4_, 2.5 CaCl_2_, and 1.3 MgSO_4_. All solutions were saturated with 95% O_2_ and 5% CO_2_, pH 7.4. Slices were incubated for at least 1 h in the recording solution before experiments.

Experiments were conducted in a submersion-type recording chamber (0.8 ml volume) perfused at 2 ml/min, and the temperature was maintained at 28±1°C using a TC-344B dual-channel heater controller (Warner Instruments, Hamden, CT). Field excitatory postsynaptic potentials (fEPSPs) were evoked in CA1 by electrical stimulation (0.05 Hz) of the Schaffer collateral pathway with a monopolar stimulating electrode filled with aCSF and a glass recording electrode filled with 1 M NaCl, both positioned in the middle third of stratum radiatum in the CA1 region. The recording and stimulating electrodes were placed at the same depth (60–100 µm), and the distance between them was kept constant. Recordings were performed with a MultiClamp 700A amplifier (Molecular Device, Sunnyvale, CA), and output signals were filtered at 3 kHz. Data were digitized (5 kHz sampling frequency) and analyzed online using a macro written in IgorPro (Wavemetrics, Portland, OR). fEPSP slopes (mV/ms) were recorded with stimulus intensities yielding 40–50% of the maximum response. After at least 15 min of stable baseline recordings in solution containing 0.01% DMSO (vehicle for E2 perfusion), 1 nM of E2 was bath applied. LTP was induced with high frequency stimulation (2 trains of 100 stimuli at 100 Hz, separated by 20 s) after 20 min of stable baseline recordings in the solution containing 1 nM E2.

### Statistical Analysis

Data are expressed as mean ± SEM. Changes in body weights over 6 months after OVX were analyzed by repeated measures ANOVA with Newman-Keuls post-hoc comparison. Loss of body weight after ischemia/sham operation (calculated by subtracting body weight on day 7 after surgery from pre-ischemia body weight) was tested by 2 way ANOVA (ischemia x hormone treatment) followed by Newman-Keuls post-hoc tests. Cell count data were first analyzed using two-way ANOVA (ischemia×hormone treatment). If significant main effects or interactions were found, data were further evaluated as follows. 1) Cell counts in sham groups were tested by Mann Whitney U-test (sham vehicle vs. sham E2). Because they did not differ, the data were combined into a single sham group. 2) Cell counts in ischemic rats relative to sham were tested using one-way ANOVA (sham vs. ischemia + vehicle vs ischemia + E2) followed by Tukey HSD. For electrophysiological data, the averaged responses for 5 min baseline recording (last 5 min before E2 infusion or LTP induction), and the averaged responses during 25–30 min after the start of E2 application or complete delivery of tetanus, respectively, were analyzed using two-way ANOVA (treatment×age) followed by post-hoc tests (paired t-tests or Mann–Whitney U tests as appropriate). The magnitude of effect was calculated as the percentage change between the two time periods described above and tested by Mann–Whitney U tests. A two tailed p<0.05 was considered significant.

## Results

### Body Weight

**Figure 1:**
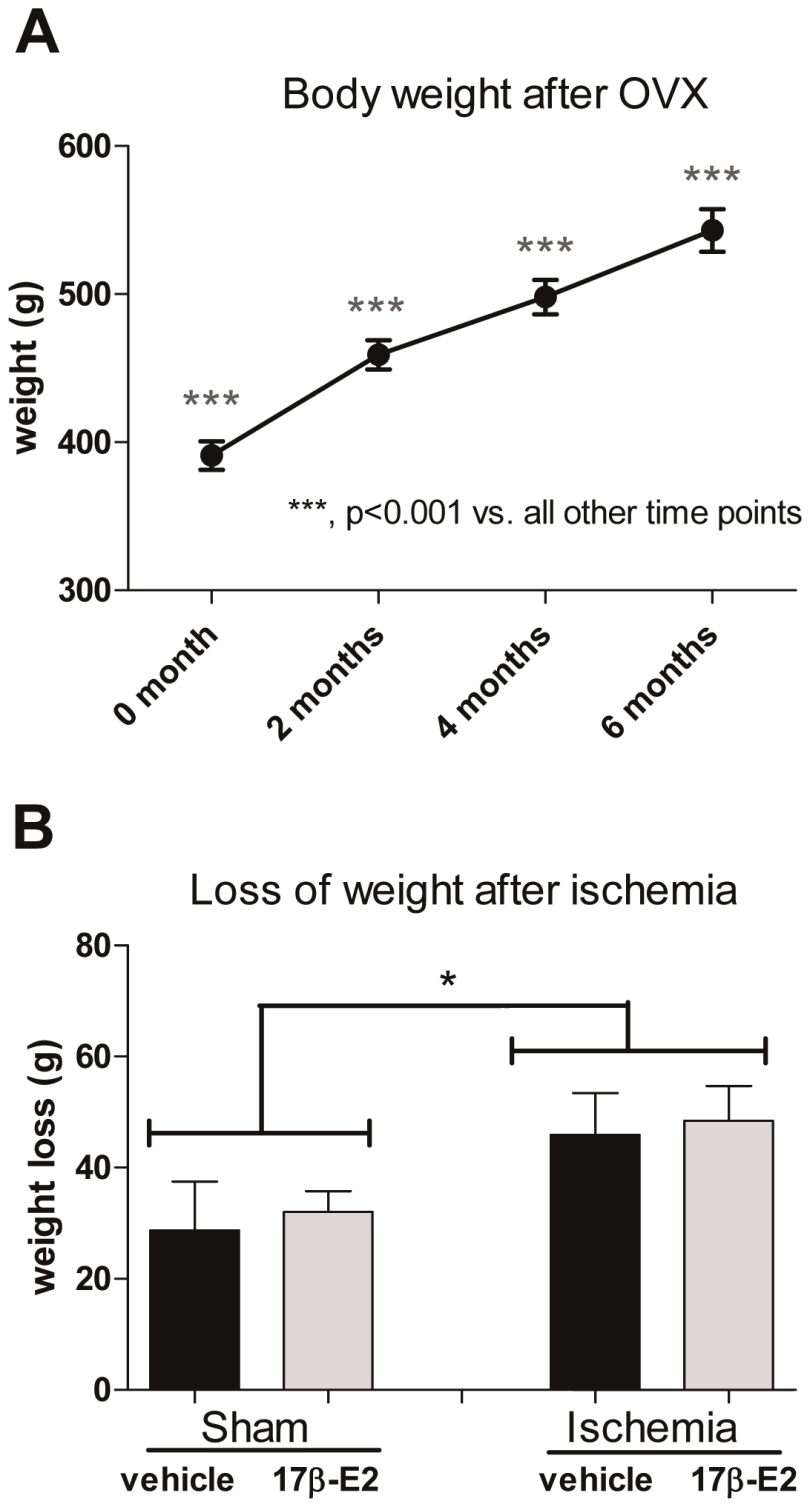
Body weight increased significantly after OVX. Panel A: The body weight of middle-aged females rats over 6 months after OVX. Panel B: The difference in body weight measured at the time of ischemia and 7 days later, when animals were killed for histological evaluation. Entries are means ± SEM. ***, *p<*0.001 vs. all other time points; *, *p<*0.05.

Changes in body weight after OVX were monitored for 6 months to confirm physiological effects of E2 deprivation over time. As shown in Fig. 1A, all animals gained weight after OVX (*F* = 148.2, p<0.0001). Average body weight was 391±10 g at the time of OVX and increased to 459±10 g (16% increase), 498±12 g (27% increase) and 543±14 g (39% increase) at 2, 4 and 6 months after OVX, respectively. Body weights at each time point differed significantly (p<0.001 for all time points). We also calculated the loss of weight due to surgery by subtracting body weight on day 7 after surgery from pre-ischemia body weight. Animals in all groups significantly lost weight after ischemia/sham operation (Fig. 1B). There was a significant main effect of ischemia (*F* = 4.38, p<0.05), with ischemic rats losing more weight than sham-operated rats, but not of hormone treatment (*F* = 0.14, p<0.91), indicating that acute E2 administration did not affect weight loss after ischemia/sham operation.

### CA1 Pyramidal Cell Survival

**Figure 2:**
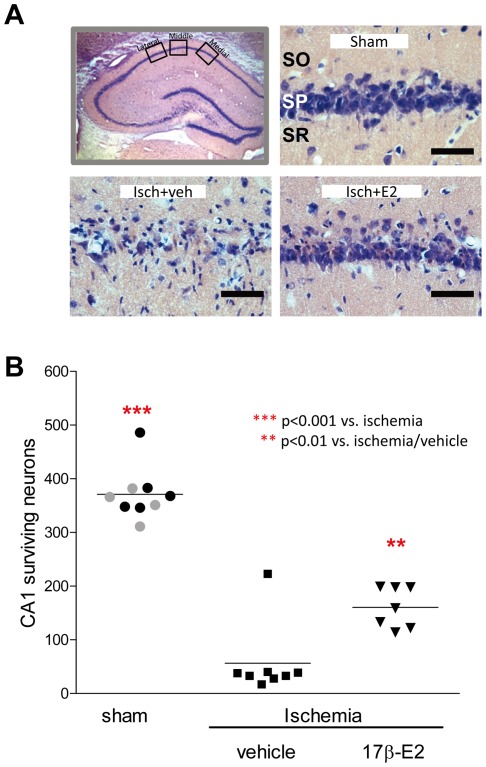
E2 given intraventricularly affords neuroprotection in aged female rats 6 months after OVX. Aged female rats were subjected to sham surgery or global ischemia 6 months after OVX. Panel A: Surviving CA1 pyramidal neurons were counted bilaterally in 3 sectors (250 µm×250 µm each) of 4 sections of the dorsal hippocampus (top left). Representative photomicrographs of neurons in dorsal CA1 one week after sham or ischemia (Isch) surgery in rats infused immediately with vehicle (veh) or 2.25 µg of E2. *Scale bars,* 60 µm. SO, stratum oriens; SP, stratum pyramidale; SR, stratum radiatum. Panel B: Surviving pyramidal neurons in the CA1 subfield of the hippocampus were quantified 7 days after ischemia. Because there were no significant differences in cell counts in vehicle (black circles) and E2 (gray circles) treated sham rats, data were combined and shown as a single sham group. Data are means ± SEM and represent the grand sum of neurons in all 3 counting sectors from all 4 sections. ***, *p<*0.001 versus ischemia; **, *p<*0.01 versus ischemia/vehicle.

For sham-operated animals, 2-way ANOVA followed by post-hoc testing indicated that there was no significant hormone effect (z = −1.23, p = 0.29). Therefore, the data were combined into a single sham group for illustration in Fig. 2. Global ischemia produced massive CA1 pyramidal cell death in all ischemic animals relative to sham-operated rats, indicated by a significant main effect of ischemia (*F = *124.5, p<.0001). There was also a significant interaction between ischemia and hormone (*F*  = 11.9, p<.001). Post-ischemic infusion of E2 into the ventricle significantly increased the number of surviving CA1 neurons (*F* = 77.5, p<0.001) in ischemic animals (45% survival in hormone-treated rats versus 15% in controls, p<0.003), indicating that post-ischemic E2 administration reduces CA1 cell death in old OVX female rats after 6 months of hormone deprivation. This level of protection is similar to that reported in our previous study, where the same dose of E2 rescued 52% of CA1 neurons in middle-aged rats OVX for 2 months [19]. Therefore, we analyzed whether CA1 survival rates are significantly different between middle-aged females OVX for 2 (data from ref 19) or aged females OVX for 6 months (Fig. 2). Mann-Whitney U tests revealed no significant differences (z = −1.22, p = 0.25), which suggests this dose of E2 rescues CA1 neurons to the same extent at both OVX intervals.

Because systemic injection of hormone is more clinically relevant than intraventricular infusion and some studies showed subcutaneous injection of E2 after ischemia is neuroprotective in young and middle-aged females [19], [24], [25], we injected a separate group of 6 month OVX rats (n = 8 ) subcutaneously with 100 µg/kg of E2. Only 17% of CA1 pyramidal neurons survived in these E2-treated ischemic rats (data not shown), which was significantly different from sham (p<0.001) and ischemic rats infused intraventricularly with E2 (p<0.01), but not from vehicle-treated ischemic animals (p = 0.99).

### CA3 and CA4 Pyramidal Cell Survival

**Figure 3:**
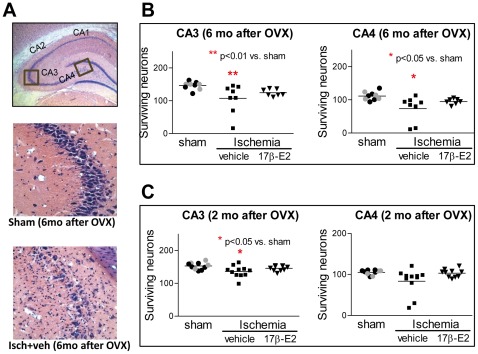
Global ischemia causes loss of CA3/CA4 pyramidal neurons in aging hippocampus Aged female rats were subjected to sham surgery or global ischemia 2 or 6 months after OVX. Because there were no significant differences in cell counts in vehicle (black circles) and E2 (gray circles) treated sham rats, data were combined and shown as a single sham group. Panel A: Surviving CA3 and CA4 pyramidal neurons (250 µm×250 µm) were counted bilaterally in one sector of 4 sections of the dorsal hippocampus (top panel). Representative photomicrographs of neurons in the CA3 and CA4 subfield in sham (middle panel) and ischemia+vehicle (bottom panel) groups. Panel B: Surviving CA3 (left) and CA4 (right) neurons in females OVX for 6 months and assessed at 1 week after sham or ischemia surgery and treated intraventricularly with vehicle or 2.25 µg of E2. Panel C: Surviving CA3 (left) and CA4 (right) neurons in females OVX for 2 months and assessed 1 week after sham or ischemia surgery and treated intraventricularly with vehicle or 2.25 µg of E2. Data are means ± SEM. *, p<0.05 versus sham.

We observed substantial CA3 and CA4 cell loss in some sections from 6 month OVX animals subjected to global ischemia. As shown in Fig.3, ischemia produced moderate (about 30% compared to sham), but significant pyramidal cell loss in vehicle-treated ischemic animals in both CA3 (*F* = 7.13, p<0.01) and CA4 (*F* = 5.86, p<0.05) areas. There were no differences in cell counts in vehicle- and E2-treated sham rats in CA3 (z = −0.37, p = 0.73) or CA4 (z = −0.25, p = 0.91). Thus, sham data were combined and used for post-hoc tests. Infusion of E2 tended to reduce neuronal loss to about 10–15%, but the effect of E2 was not statistically significant (Fig. 3B). To determine whether CA3/CA4 cells are vulnerable to ischemia in middle-aged females subjected to shorter periods of hormone deprivation, we counted CA3 and CA4 pyramidal cells from middle-aged, retired breeders subjected to ischemia or sham surgery 2 months after OVX and treated intraventricularly with vehicle or E2 as described in Methods. Ischemia did not affect CA4 cell counts (*F* = 2.18, p<0.15), but significantly reduced CA3 pyramidal neurons (about 12% cell loss, *F* = 6.13, p<0.05) in these animals (Fig. 3C). There was a tendency for E2 to attenuate CA3 pyramidal cell death in ischemic rats, but the effect was not significant.

### Synaptic Physiology

**Figure 4:**
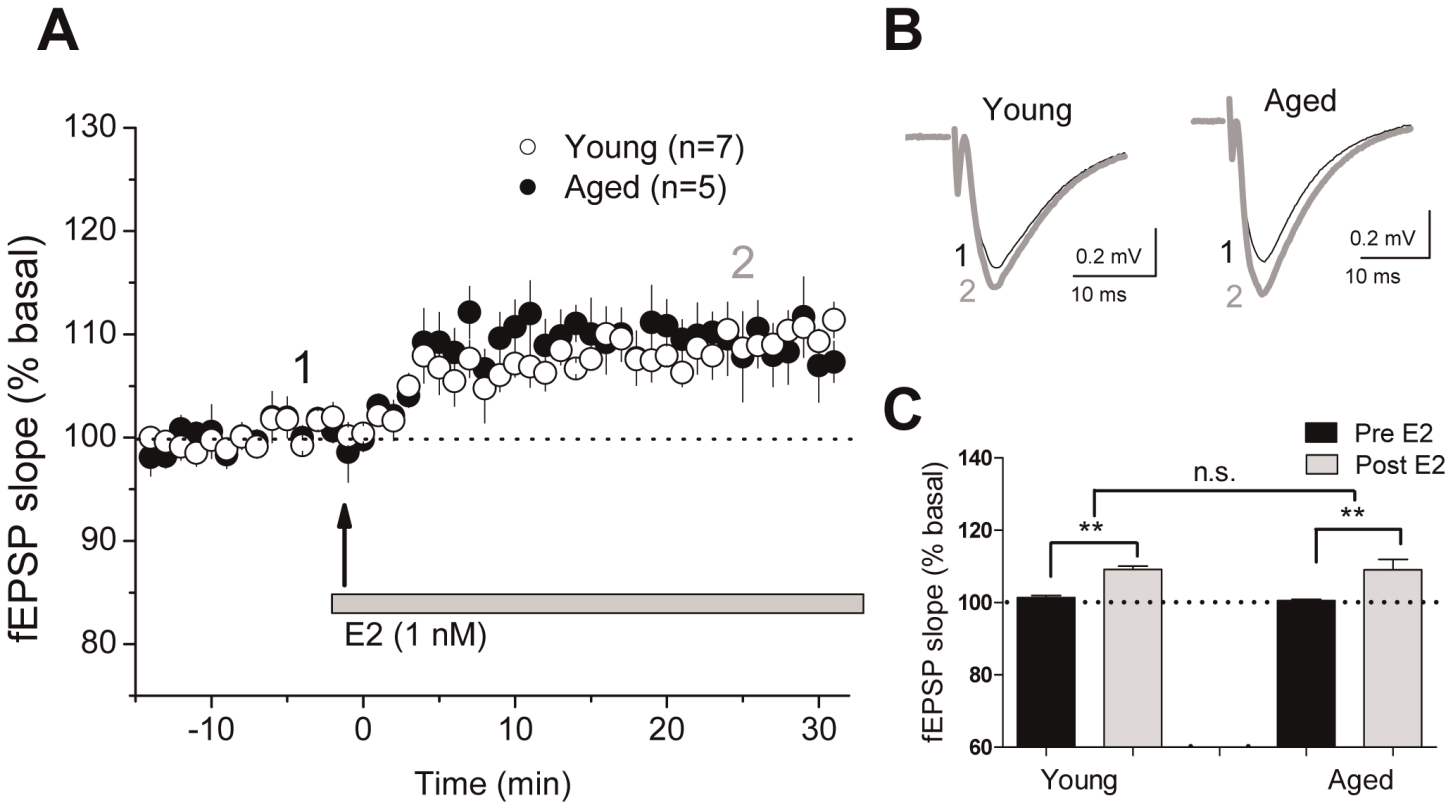
CA1 synapses in the aging hippocampus retain responsiveness to E2 after long-term hormone deprivation. Evoked fEPSPs were measured in acute hippocampal slices from aged (15–18 mo, 6 months after OVX) and young (2 mo, 7–10 days after OVX) females. E2 (1 nM) was bath applied after 15 min stable baseline. Panel A: E2 facilitated fEPSPs in young (open circles) and aged (black circles) females with short latency. Panel B: Representative fEPSPs from young (n = 7 slices from 6 rats) and aged (n = 5 slices from 4 rats) rats before and after E2 application. Panel C: The averaged responses for the last 5 min baseline recording and the averaged responses during 25–30 min after the start of E2 application. Data are means ± SEM. n.s., not significant. **, *p*<0.01.

We recorded fEPSPs at CA3-CA1 synapses elicited by Schaffer collateral stimulation in acute hippocampal slices prepared from aged (15–18 mo, 6 months after OVX) and young adult (2 mo, 7–10 days after OVX) females. Bath application of E2 (1 nM) significantly enhanced synaptic responses (*F* = 43.1, p<0.001, Fig.4) in slices from both young (110.2±1.0% of baseline, 7 slices from 6 animals, p<0.01 vs. baseline) and aged (109.1±2.4% of baseline, 5 slices from 4 animals, p<0.01 vs. baseline) OVX rats with a similar latency (approximately 5–7 min) and magnitude measured 25–30 min after the start of E2 application.

**Figure 5:**
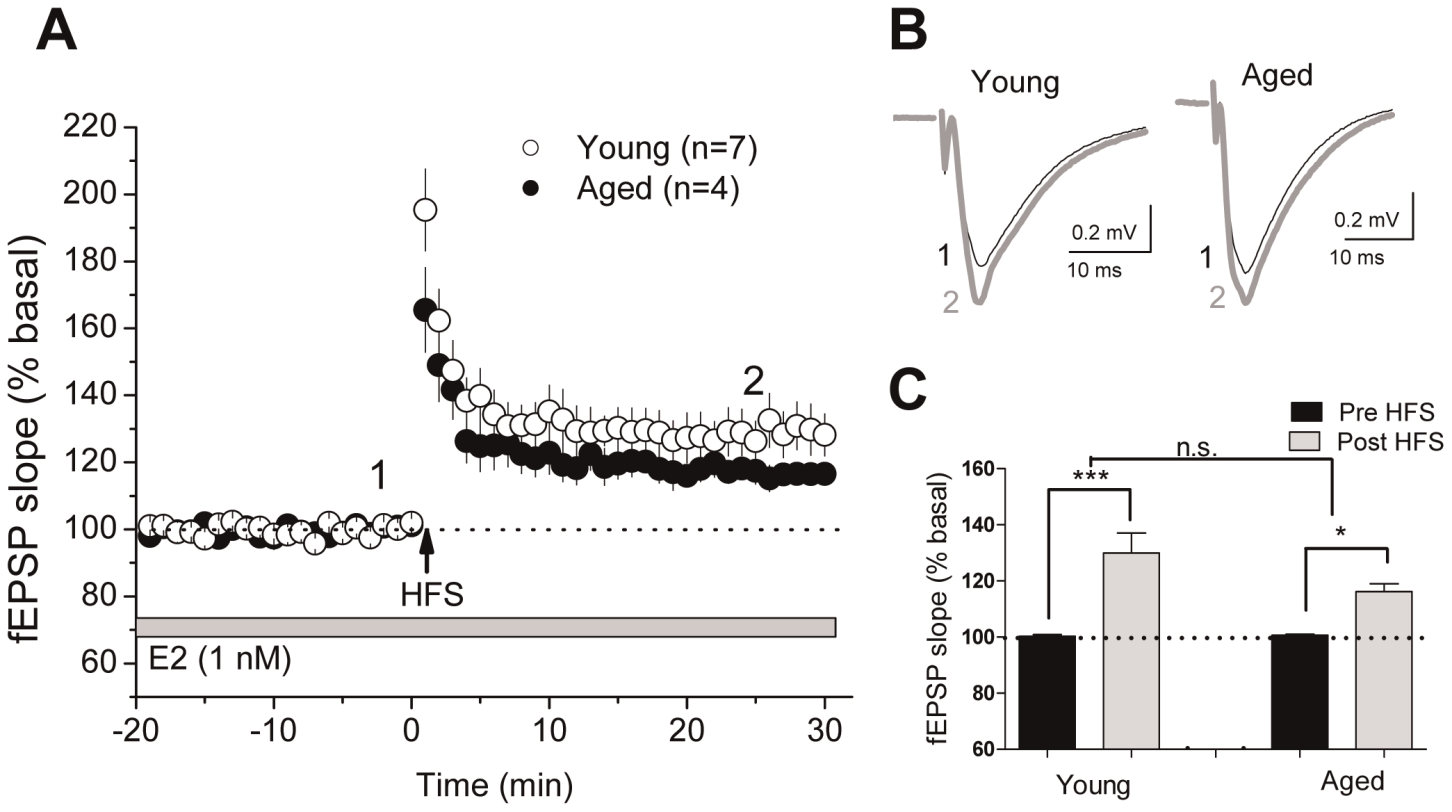
Aged hippocampus exhibits normal LTP. Acute hippocampal slices were prepared from aged (15–18 mo, 6 months after OVX) and young (2 mo, 7–10 days after OVX) females. Panel A: High frequency stimulation (HFS, 2 trains of 100 stimuli at 100 Hz) was applied after 20 min baseline in the presence of E2. LTP was induced in slices from both young (open circles) and aged (black circles) rats. Panel B: Representative fEPSP traces from young (n = 7 slices from 6 rats) and aged (n = 4 slices from 4 rats) rats before and after HFS. Panel C: Averaged responses for the last 5 min baseline recording and the averaged responses during 25–30 min after complete delivery of tetanus. The magnitude of LTP between the two groups was not significantly different. Data are means ± SEM. n.s., not significant. *, *p*<0.05, ***, *p*<0.001.

Because we had very few old animals for electrophysiological studies, the LTP experiments were carried out in slices exposed to E2, after they had achieved a stable fEPSP. In slices from young females recorded under our conditions, 1 nM E2 does not modify LTP induced by high frequency stimulation (data not shown). fEPSPs were significantly potentiated after high frequency stimulation (*F* = 21.8, p<0.01, Fig.5) in slices from young (129.9±6.1% of baseline, 7 slices from 6 animals, p<0.001 vs. baseline) and aged (116.2±2.1% of baseline, 4 slices from 4 animals, p<0.05 vs. baseline) OVX rats. The magnitude of LTP in aged OVX animals was not significantly different from that in young OVX animals.

## Discussion

The current study addressed the efficacy of E2 to protect CA1 pyramidal neurons in old, long-term hormone deprived female brain in a clinically relevant model of global ischemia, examining whether a high dose of E2 administered immediately after ischemia is neuroprotective in aging hippocampus. Here, we report that a single dose of E2 infused intraventricularly immediately after ischemia ameliorates CA1 pyramidal neuron death in 15–18 month female rats after 6 months of hormone withdrawal. We also show that bath application of E2 facilitates fEPSPs at CA1 synapses and that high frequency stimulation induces LTP in acute slices from aged females OVX for 6 months to the same extent as in young adult females. These results provide strong evidence that the aging female hippocampus retains responsiveness to the neuroprotective actions of high doses of E2 even after prolonged periods of hypoestrogenicity.

### E2 is Neuroprotective in Aged, Long-term Hormone-deprived Female Rats

The finding that E2 retains its ability to protect the aging hippocampus from ischemic injury is consistent with our previous report that post-ischemic infusion of E2 and some estrogen analogs that do not bind classical estrogen receptors (ERs), such as the GPR30 agonist G1 [26] and the membrane-associated ER agonist STX [27], [28], attenuate ischemia-induced cell death in middle-aged females OVX for 2 months before experimentation [19]. Thus, our data provide additional evidence that post-ischemic infusion of E2 is neuroprotective in old rats even after extended periods of hypoestrogenicity. In contrast, systemic injection of E2 (100 µg/kg) in a dose that rescued CA1 neurons in middle-aged rats OVX for 2 months [19] was not effective in rats that were OVX for 6 months. The different outcomes between intraventricular and systemic E2 treatment may relate to the dramatically increased body weights observed in 6 month OVX females. As shown in Fig. 1, weights in rats OVX for 6 months increased by almost 40%, and most of the weight gain is due to increased adiposity. Thus, one possible explanation is that increased body fat reduces or slows the ability of subcutaneously injected E2, which is highly lipophilic, to reach the target brain areas in high concentrations. Supporting this idea, when E2 is directly administered into the brain ventricles, CA1 pyramidal cell survival rates in ischemic females OVX for 6 and 2 months (45% and 52% survival, respectively) are not statistically different. Because obesity worsens apoptosis and neuroinflammation after hypoxic ischemia [29], this may also contribute to the reduced efficacy of systemically administered E2.

In contrast with our results, several investigators report that E2 loses its ability to protect neurons from focal and global ischemia if hormone treatment is not initiated shortly after OVX [10], [11], [12], [13], [14], even in young animals. Several possible explanations may account for the different findings. First, three of the studies examined outcomes after focal ischemia [10], [12], [13] induced by middle cerebral artery occlusion (MCAO), whereas we induced transient global ischemia. The focal and global ischemia models differ in several ways, including the nature of brain injury and the specific brain regions involved. Thus, sensitivity to exogenous E2 treatment after prolonged OVX intervals may depend on the type of brain damage and the affected brain region.

Yet the most likely explanation for the differing outcomes between our work and others may be the dose and timing of E2 administration [30]. Studies that found no beneficial effects of E2 after long-term loss of ovarian hormones administered E2 chronically for days or weeks prior to ischemia using methods that maintain physiological levels of E2 (10–80 pg/ml) [10], [11], [12], [13], [14]. These studies attempt to mimic circulating hormone levels to test the critical period hypothesis in a model similar to the continuous, long-term HT used in menopausal women. This form of neuroprotection is mediated through slow genomic actions of E2 [31], involving regulation of various pro- and anti-apoptotic genes [32], [33], and classical ERs play a key role. For example, MCAO studies in mice with targeted deletion of classical ERs [34] and in rats treated with an ER antagonist [11], [35] or selective agonists in global [36] and focal ischemia [37] show that classical ERs, in particular ER-α, are necessary to protect neurons against ischemia-induced brain injury. Indeed, a recent study reports degradation of ER-α as a key mechanism for the loss of E2 neuroprotection when global ischemia is induced in very old rats or in younger rats subjected to long periods of hormone deprivation, suggesting that the loss of E2 neuroprotection is associated with the degradation of hippocampal ER-α in aged and long-term hormone withdrawn female rats [14].

In contrast, the present and earlier studies that report beneficial effects of E2 used a high dose of hormone given immediately after [19] or 30 min before [15] ischemia. These studies test the efficacy of E2 as a neuroprotective strategy after long-term loss of ovarian hormones using a supraphysiological dose of E2 administered just before or after the ischemic event. This form of E2 action may be mediated via rapid, non-genomic, membrane-initiated E2 signaling pathways [27], [38], [39]. For example, estrogenic compounds that have no/little affinity for classical ERs [19], [40] including several non-feminizing estrogen analogs [41], are neuroprotective in focal and global ischemia. These findings suggest that neuroprotective actions of post-ischemic E2 may involve membrane-associated estrogen binding sites that are independent of ER-α and ER-β and that engage signaling pathways associated with cell membrane receptors. For instance, global ischemia transiently increases Akt phosphorylation and decreases the phosphorylation of the Akt targets, GSK3β and FOXO3A, and activates caspase-3 in the CA1 subfield shortly (1–3 h) after ischemia; acute post-ischemic E2 rapidly inhibits these early events and provides histological protection in young OVX females [42]. These findings suggest that regulation of Akt signaling in temporal proximity to an ischemic event is important for neuronal survival. There is also evidence that E2 significantly increases phospho-Akt-immunoreactivity in CA1 dendritic spines of young and aged female rats [43]. Thus, we speculate that enhanced Akt signaling may be one possible mechanism underlying the observed neuroprotective effects of E2, and that E2 can regulate these signaling cascades in the aging hippocampus even after a long period of hormone deprivation.

Accumulating evidence also indicates that acute, high-dose treatment with E2 after ischemia effectively reduces neuronal damage in permanent and transient focal ischemia in adult OVX animals [25], [44], [45], even when treatment is delayed up to 6 h after the onset of ischemia [24]. If delayed treatment is also effective in old animals subjected to global ischemia, post-ischemic E2 therapy may have great neuroprotective potential for treatment of ischemia/stroke in post-menopausal women. Intriguingly, the range of effective E2 concentrations in promoting neuronal survival after ischemic insults is very large, from physiological (100 pM) to pharmacological (50 µM) *in vitro*, and from 100 µg/kg to 1000 µg/kg *in vivo*, depending on the timing of treatment after ischemia [44]. The E2 dose used in the current study (2.25 µg or 450 µg/kg) is supraphysiological, but falls into this effective concentration range. Interestingly, concentrations of E2 in the brain, including the hippocampus, are reported to be much higher than circulating E2 [46], [47]. Locally synthesized E2 acts in a paracrine and autocrine manner [48], rapidly modulating synaptic physiology and plasticity through non-genomic actions [49], [50]. Because critical enzymes required for E2 synthesis, such as aromatase and StAR, are synaptically localized in hippocampal neurons and glial cells [51], the possibility arises that long-term OVX rats may retain the ability to produce some brain-derived E2 even after loss of circulating E2, and that E2 administered after ischemia may interact with locally synthesized E2 to protect neurons. This speculation is supported by findings indicating that brain-derived E2 plays a role in neuroprotection [52], [53], [54]. In some models, aromatase activity increases after ischemia [55], [56], and the extent of ischemic damage is greater in aromatase knockout than in wild-type females [57]. Furthermore, MCAO increases brain E2 concentrations, and co-injection of an aromatase inhibitor decreases brain E2 levels and is associated with more damage [58]. Thus, brain-derived E2 may act as a natural neuroprotective agent. However, it is obvious that hippocampal E2 alone is not sufficient to prevent brain damage induced by ischemia as all studies report significant cell death after ischemia in vehicle-treated animals.

### The Aging Hippocampus is Sensitive to E2 and can Exhibit LTP at CA1 Synapses after Long-term Hormone Deprivation

It is well known that E2 rapidly increases excitability and basal synaptic transmission at CA1 synapses, and under some circumstances enhances LTP, via non-genomic actions [59]. Although aging affects synaptic physiology [60] and the aged hippocampus may respond differently to E2 [61], in agreement with the histological outcomes, the current electrophysiological experiments demonstrate that application of E2 onto brain slices derived from old females OVX for 6 months facilitates fEPSPs at CA1 synapses. This action of E2 is indistinguishable in latency and magnitude to that recorded in slices from young rats. Moreover, slices from old, hormone-withdrawn females exhibit normal LTP in response to high frequency stimulation. Thus, the current results provide solid evidence that the aging hippocampus retains some responsiveness to E2 for at least at 6 months after OVX.

Two studies that examined the effects of long-term hormone deprivation on E2 enhancement of CA1 synaptic transmission and plasticity in aging hippocampus are consistent with the current results. The first showed that E2 treatment for 2 days in vivo increases the magnitude of LTP evoked at CA3-CA1 synapses by high frequency stimulation, spine density and synaptic currents up to 15 months after OVX [62]. The second reported that bath application of E2 enhances fEPSPs by 20%, and that stable LTP is induced by theta-burst stimulation of the Schaffer collateral pathway in hippocampal slices from 15–16 month female rats 6 months after OVX [63]. Interestingly, the latter investigators also found that OVX depresses actin signaling cascades involved in synaptic plasticity, and that acute infusion of E2 rapidly activates this pathway [64]. These findings suggest a possible mechanism underlying short latency E2 effects at CA1 synapses and provide evidence that the aging hippocampus retains the ability to activate actin signaling cascades in response to E2 after long-term loss of ovarian hormones.

The E2 concentration (1 nM) used in the electrophysiological experiments is higher than peak circulating E2 levels (40–80 pg/ml, approximately 109 pM) seen during proestrus in rats [59], but it is similar to hippocampus-derived E2 levels measured in intact females (0.5–1.7 nM) [47]. Previous studies in slices from young rodents show dose-dependent E2 enhancement of excitatory synaptic transmission at CA1 synapses both in males [63] and E2-primed, OVX females [65]. In males, infusion of E2 at concentrations of 0.01–1 nM produces a rapid, dose-dependent and reversible increase in excitatory synaptic transmission at CA3-CA1 synapses, and the effects 0.1–1 nM E2 are significantly different from those observed in 0.01 nM E2 [63]. Thus, the threshold for E2 facilitation of synaptic transmission may lie between 0.01 and 0.1 nM. Similarly, in E2-primed OVX females, bath application of 0.1 to 100 nM E2 dose-dependently potentiates excitatory postsynaptic currents within a few minutes [65]. Although we only tested 1 nM in this study, the comparable effects of E2 on enhancement of fEPSP slope in slices from young and old OVX rats suggest that a concentration-dependent E2 modulation would be observed at CA1 synapses in slices derived from old, long-term OVX females.

### Ischemia Kills Pyramidal Neurons Beyond the CA1 Subfield in Aged Rats

Another important outcome in the present study is that global ischemia causes death of pyramidal neurons in the CA3 and CA4 subfields in older females OVX for either 2 or 6 months. It is well documented that different populations of hippocampal neurons have distinct vulnerability to ischemia [66], [67], and that cell loss in CA3/CA4 does not usually occur in young rodents unless the duration of ischemia is prolonged [68]. On the other hand, age-related changes in vulnerability of CA3 pyramidal neurons to ischemia [69] have been reported. There is other evidence of age-associated increases in susceptibility to brain damage [70], [71], [72], and the aging hippocampus undergoes a variety of biochemical, structural and functional changes, including reduction of IGF-1 levels [73], decreases in dendritic spines [74] and altered ER-α distribution [61].

In a previous study, degeneration of CA3/CA4 neurons after global ischemia was observed in young female rats OVX for 10 weeks, and pretreatment with physiological levels of E2 for one week prior to ischemia did not rescue this cell loss [11]. In the present experiment, it is uncertain whether acute E2 protects CA3 and CA4 neurons from ischemia-induced cell death. Cell counts were statistically indistinguishable in sham-operated and ischemic rats treated with E2, suggesting that there might be some protection. However, the absence of a significant difference between vehicle- and E2-treated ischemic rats calls this into question. Because this pattern is the same at both OVX intervals, perhaps there is a weak protective effect that is not influenced by age or OVX duration.

### Conclusion

In conclusion, present data show that acute post-ischemic E2 infusion into the brain ventricle ameliorates ischemia-induced death of hippocampal CA1 pyramidal neurons in aged rats after 6 months of ovarian hormone withdrawal. Moreover, bath application of E2 rapidly facilitates fEPSPs at CA1 synapses in hippocampal slices from aged females OVX for 6 months with the same short latency and magnitude observed in young females. These results provide clear evidence that the aging female hippocampus retains responsiveness to certain actions of E2 even after a prolonged period of ovarian hormone deprivation. Our data further indicate that the type of ischemia as well as the dose and timing of E2 administration are important factors that determine E2 responsiveness in the aging brain. These findings have clinically important implications for potential treatment of global ischemia in post-menopausal women.
